# Diversity and prevalence of gastrointestinal parasites in farmed pigs in Southeast Gabon, Central Africa

**DOI:** 10.14202/vetworld.2019.1888-1896

**Published:** 2019-12-02

**Authors:** Gael Darren Maganga, Linda Bohou Kombila, Larson Boundenga, Ivan Cyr Moussadji Kinga, Judicael Obame-Nkoghe, Herve Tchoffo, Oubri Bassa Gbati, Julius Awah-Ndukum

**Affiliations:** 1 Centre International de Recherches Médicales de Franceville, BP 769, Franceville, Gabon; 2 Département de Zootechnologie, Institut National Supérieur d’Agronomie et de Biotechnologies (INSAB), BP 901, Franceville, Gabon; 3 Département de Biologie, Université des Sciences et Techniques de Masuku (USTM), BP 901, Franceville, Gabon; 4 Animal Physiology and Health Research Unit, Faculty of Agronomy and Agricultural Sciences, University of Dschang, P.O. Box 188, Dschang, Cameroon; 5 Laboratoire de Parasitologie et de Mycologie de l’EISMV, BP 5077, Dakar, Sénégal; 6 Department of Microbiology and Infectious Diseases, Ecole des Sciences et de Médecine Vétérinaire, Université de Ngaoundéré, Ngaoundéré, Cameroun

**Keywords:** diversity, Gabon, gastrointestinal parasites, pigs, prevalence

## Abstract

**Background and Aim:**

Gastrointestinal infestations caused by intestinal parasites are the most important diseases and the most common in pigs in the tropics. These parasites are often associated with a huge economic loss. This study aimed to assess the diversity and prevalence of gastrointestinal parasites in farmed pigs from Haut-Ogooue Province, in South East Gabon.

**Materials and Methods:**

From March 2018 to July 2018, 156 samples of pig feces collected from nine different farms were analyzed under light microscopy. The identification of eggs, cysts, and oocysts in fecal samples was done using two qualitative techniques: Flotation and sedimentation.

**Results:**

After examination, the results obtained revealed an overall infestation level of 98.7% (154/156). We found ten parasite types with infestation levels that varied from species: *Balantidium coli* (120/156), *Oesophagostomum* spp. (100/156), *Isospora suis* (102/156), *Ancylostoma* spp. (17/156), *Trichostrongylus* spp. (28/156), *Hyostrongylus* spp. (13/156), *Strongyloides* spp. (7/156), *Ascaris suum* (8/156), *Globocephalus* spp. (1/156), and *spirurida* (1/156). The study of risk factors revealed that factors such as sex, age, and physiological condition may influence the diversity and level of infestation of animals by gastrointestinal parasites.

**Conclusion:**

For better prevention of parasitism in these farms, it would be interesting to implement health monitoring and to ensure good hygiene. Finally, further studies would be needed to better evaluate the distribution of these parasites in Gabon and the involvement of these animals in the transmission cycle of parasitic zoonoses.

## Introduction

In Gabon, animal husbandry occupies a marginal place in its economy. Indeed, the latter would represent <1% in the gross domestic product. Therefore, it is not a creator of wealth. Even though Gabon imports most of its products from poultry, pigs, or beef from neighboring countries [1,2], the fact remains that short-cycle animal rearing activities such as pig are emerging in some of its localities. For example, poultry and pig farming accounts for 80% of the peri-urban rearing activities of Libreville, the capital. A study carried out in the peri-urban areas of Libreville revealed that, based on the number of farms, pig farms came in second after the flocks of laying hens, with a herd estimated at 1207 [3]. For smallholders, pig farming offers real potential for economic gain, considering factors such as feed conversion efficiency, high fecundity (very prolific species), and short generation intervals [4]. In the peri-urban area of Libreville, the economic weight of the activity is estimated at 12,005,000 FCFA of annual income [3]. However, these small farmers face a number of obstacles, the most important of which are parasitic diseases that induce high mortalities rate and low weight gain ­compared to industrialized production systems, leading to economic losses [4]. The main difficulties in these farms include hygiene and prophylaxis. In fact, the pig breeding, like that of other animals, is limited, among others, by the negative effect of parasites and diseases on production [5].

Parasitic infestations play a central role in the biodiversity of ecosystems and communities because of their impact on the dynamics of population growth and regulation [6,7]. Among the parasitic helminths, nematodes are considered to be the most important around the world [8]. Gastrointestinal infestations caused by these parasites are the cause of the most important and most common diseases in pigs in the tropics [9]. These parasitic diseases are most often associated with huge economic losses due to the fact that they will reduce litter sizes, causes poor growth, or reducing the rate of weight gain [10]. Previous studies on helminth infestations in pigs have reported the presence of several species of parasites such as *Ascaris suum*, *Strongyloides ransomi*, *Trichuris suis*, and *Oesophagostomum* spp. in several African countries [8,11,12]. These parasites are known to have deleterious effects on animal health.

This study aimed to determine the diversity and prevalence of gastrointestinal parasites affecting farmed pigs in Haut-Ogooué Province, Southern Gabon, because, to date, pig farming is expanding in this region. In addition, no information is available in the country on the nature and prevalence of gastrointestinal parasites in pigs.

## Materials and Methods

### Ethical approval and informed consent

For this study, fecal sample collection from pigs was approved by la Direction Provinciale de l’Elevage et de l’Agriculture. Oral consent was obtained from all owners of pig farms.

### Study area

**Figure-1:**
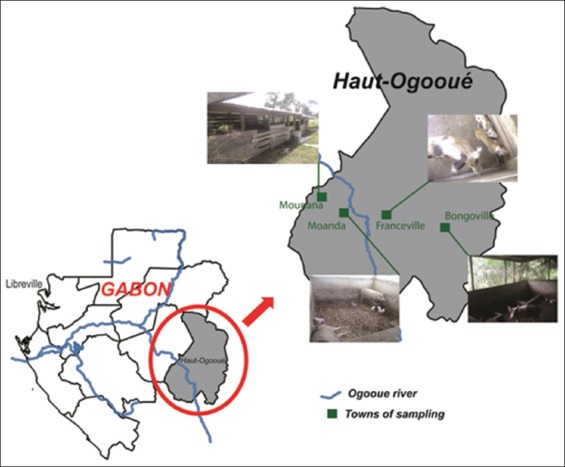
Location of sampling sites for pig feces. This figure shows the province in which the study took place and the cities where animal feces were collected. [Source: The authors made the figure with the help of Illustrator CS6 software].

The study was conducted from March 2018 to July 2018, in nine pig farms located in four towns (Bongoville, Franceville, Moanda, and Mounana) in the Haut-Ogooué Province in Southeastern Gabon (Figure-1). These are small pig farms owned by individuals who sell pig meat in markets or restaurants. The study area is characterized by an equatorial climate divided into four seasons that alternate between rainy season and dry season. There is no inventory of pig herds in this region.

### Sampling

Farms were selected on the basis of an agreement from the farm manager to participate in the study after presenting the objectives of the study. Pigs on each farm were selected for accessibility and regardless of age, sex, and general body condition. No anesthetic or other chemical treatment was administrated before feces collection. Fresh feces from 156 pigs, including 74 males and 82 females, were collected from the ground and after defecation or from the rectum. The feces of each individual were harvested using a well-identified sampling pot. The samples collected were stored refrigerated in a cooler and transported to the parasitology laboratory of the Centre International de Recherches Médicales de Franceville (CIRMF) to be analyzed the same day.

Data on sex, age, race, general body condition, physiological condition, breeding system, and livestock management were recorded using a survey questionnaire.

### Parasitological analysis

The identification of eggs, cysts, and oocysts in fecal samples was done using two qualitative techniques: Flotation, using Willis liquid (aqueous solution of sodium chloride saturation of 1.2 specific gravity), and sedimentation, performed according to Gillespie [13]. The parasitic forms were observed at 40× and 100× using a light microscope equipped with a camera (Leica, Microsystems). The identification of parasites was based on the morphology of the wall and nucleus of the egg or cyst [14]. The identification of parasitic forms under the microscope was carried out with the identification keys of Herbert [15]. Confirmation of the identification of eggs and cysts of parasites was made by the Laboratory of Parasitology and Mycology de l’Ecole Inter-Etats des Sciences et Médecine Vétérinaires (EISMV) of Dakar.

### Statistical analysis

The statistical analyses were performed using R software (version 3.1.0 R Foundation for Statistical Computing, Vienna, Austria). The parasite infestation rates based on sex, age, race, the general body condition, the physiological condition, and the breeding system were calculated and compared using the Chi-square test. The differences were considered statistically significant at the 5% significance level.

## Results

### Diversity and prevalence of gastrointestinal parasites

**Figure-2:**
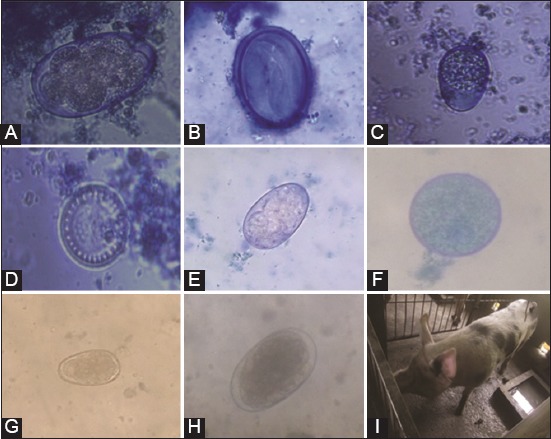
The eggs of the gastrointestinal parasites identified. This image realized with the software illustrator CS6 shows the different parasites observed in our study. (A) *Ancylostoma* spp., (B) *Spirurida*, (C) *Isospora suis*, (D) *Ascaris suum*, (E) *Globocephalus* spp., (F) *Balantidium coli*, (G) *Trichostrongylus* spp., (H) *Oesophagostomum* spp., (I) host species.

From a total of 156 fecal samples, analyses revealed an overall prevalence of 98.7% (154/156). The pigs were found to be positive for *Ancylostoma* spp., *Oesophagostomum* spp., *A. suum*, *Strongyloides* spp., *Trichostrongylus* spp., *Hyostrongylus* spp., *Globocephalus* spp., and *spirurida*, as well as for the protozoa *Balantidium coli* and *Isospora suis* (Figure-2).

In pigs, *Oesophagostomum* spp. was most common with an overall prevalence of 50% followed by *Trichostrongylus* spp. and *Ancylostoma* spp. whose overall infestation rates were 17.94% and 10.89%, respectively. For other helminths, the overall prevalence ranged from 0% to 8.313% (Table-1). For protozoan cysts excreted by pigs, *B. coli* had a higher overall prevalence at 76.76% followed by *I. suis* (65.38%) (Table-1). In addition, our results showed that all the parasites identified were relatively common in all investigated pig farms.

**Table-1:** Parasitic diversity and global prevalences.

Parasites	Global prevalence’s (%)	Sex				Comparison						Ages				Comparison					
			
		Males		Females		χ^2^	p-value	df	M+	F+	S+	Juveniles		Adults		χ^2^	p-value	df	J+	A+	S+
			
		n/74	p-value (%)	n/82	p-value (%)							n/118	p-value (%)	n/38	p-value (%)						
Helminths																					
*Oesophagostomum* spp.	50	52	70.2	48	58.5	0.34	0.55	1	+		-	81	68.6	19	50	0.73	0.39	1	+		-
*Ancylostoma* spp.	10.89	8	10.8	9	10.9	0	0.1	1		+	-	11	9.3	6	15.7	0.48	0.49	1		+	-
*Trichostrongylus* spp.	17.94	10	13.5	18	21.9	0.89	0.35	1		+	-	20	16.9	8	21	0.06	0.81	1		+	-
*Strongyloides* spp.	4.48	5	6.7	2	2.4	0.73	0.4	1	+		-	7	5.9	0.0	0	1.07	0.30	1	+		-
*Ascaris suum*	3.84	4	5.4	4	4.8	0	1	1	+		-	8	6.7	0	0	1.35	0.25	1	+		-
*Hyostrongylus* spp.	8.33	3	4.0	10	12.1	1.97	0.16	1		+	-	6	5.0	7	18.4	3.94	0.047	1		+	+
*Spirurida*	0.64	0	0.0	1	1.2	0	1	1		+	-	1	0.8	0	0	0	1	1	+		-
*Globocephalus* spp.	0.64	0	0.0	1	1.2	0	1	1		+	-	1	0.8	0	0	0	1	1	+		-
Protozoa																					
*Balantidium coli*	76.92	56	75.6	64	78.0	32.18	1.4^e-08^	1		+	+	87	73.7	33	86.8	0.20	0.65	1		+	-
*Isospora suis*	65.38	55	74.3	47	57.3	0.32	0.372	1	+		-	84	71.1	18	47.3	1.26	0.26	1	+		-

This table shows the distribution of parasites by sex and age. M+=Males having had the highest prevalence, F+=Females having had the highest prevalence, D+=Juveniles having had the highest prevalence, A+=Adults having had the highest prevalence, S+=Indicating a statistically significant difference

### Factors for variation of parasitic infestation

First, regarding the effect of sex on parasite infestation, a very marked difference between the two sexes was observed only for *B. coli* (χ^2^=32.18, df=1, p=1.4^e−08^). Indeed, we observed a significantly high prevalence in females (78%) than in males (75.6%). However, for the other parasite types, no significant difference was observed, despite differences in prevalence observed (Table-1). Furthermore, the diversity of parasites found was greater in females (10) than in males (8), although this difference was not statistically significant (χ^2^=3^e−04^, df=1, p=0.98).

Second, our results showed that overall parasite infestation rates are higher in young pigs with reference to the adults. However, age had a statistically significant influence on *Hyostrongylus* spp. infestation (χ^2^=3.94, df=1, p=0.047). Indeed, adults had a significantly higher prevalence (18.4%) of *Hyostrongylus* spp. than juveniles (5%) (Table-1).

In addition, our results revealed that imported breeds appeared to have higher infestation rates than local breeds. However, no significant difference was observed (Table-2).

**Table-2:** Prevalence of parasites types according to breed and general body condition of pigs.

Parasites	Breed										General body condition									
	
	Imported breed		Local breed		Comparison						Thin		Overweight		Comparison					
					
	n/42	p-value (%)	n/21	p-value (%)	χ^2^	p-value	df	IB+	LB+	S+	n/66	p-value (%)	n/90	p-value (%)	χ^2^	p-value	df	T+	O+	S+
*Oesophagostomum* spp.	22	52.4	12	57.1	0	1	1		+	-	49	74.24	51	78.4	0.85	0.36	1		+	-
*Ancylostoma* spp.	4	9.52	2	9.5	0	1	1	-		-	4	6.06	11	12.2	0.81	0.37	1		+	-
*Trichostrongylus* spp.	11	26.19	3	14.2	0.35	0.58	1	+		-	8	12.12	12	13.3	0	1	1		+	-
*Strongyloides* spp.	3	7.14	3	14.2	0.14	0.71	1		+	-	2	3.03	4	4.4	2^e-04^	0.98	1		+	-
*Ascaris suum*	0	0	0	0	-	-	-			-	1	1.51	4	4.4	0.29	0.6	1		+	-
*Hyostrongylus* spp.	7	16.66	1	4.7	0.63	0.43	1	+		-	7	10.6	2	2.2	3.021	0.08	1	+		-
*Spirurida*	1	2.38	0	0	0	1	1	+		-	0	0	1	1.11	0	1	1		+	-
*Globocephalus* spp.	1	2.38	0	0	0	1	1	+		-	0	0	1	1.11	0	1	1		+	-
*Balantidium coli*	33	78.57	18	85.7	4^e-04^	0.98	1		+	-	52	78.78	68	75.5	0.002	0.96	1	+		-
*Isospora suis*	18	42.85	11	52.3	0.037	0.85	1		+	-	43	65.15	59	65.5	0	1	1		+	-

IB+=Imported breed having had the highest prevalence, LB+=Local breed having had the highest prevalence, T+=Thin having had the highest prevalence, O+=Overweight having had the highest prevalence, S+=Indicating a statistically significant difference

The result of parasitic infestation rate according to the general body condition of the animals showed no significant differences between thinned and overweight pigs. However, it would appear, irrespective of the breed, that pigs in overweight had higher infestation rates than the thinned pig, for most of the identified parasites (Table-2).

Regarding the effect of the breeding system, the results showed no statistically significant difference between the extensive and semi-intensive systems, regardless of the type of parasite (Table-3). However, pigs reared in semi-intensive mode had the highest infestation rates compared to extensively raised pigs, except for *Oesophagostomum* spp. and *B. coli* (Table-3). In addition, by comparing the infestation rate to the number of individuals in a box, the results indicated that there was no significant difference between pigs raised at least two in a box and pigs reared alone in a box, except for *Strongyloides* spp. which was significantly more prevalent in pigs reared individually in a box (χ^2^=15.1, df=1, p=0.0001) (Table-3).

**Table-3:** Prevalence of the types of parasites according to the breeding system and the number of animals per box.

Parasites	Breeding system				Comparison						Number of pig per box				Comparison					
			
	Extensive		Semi-intensive		χ^2^	p-value	df	E+	SI+	S+	1 per box		>2 per box		χ^2^	p-value	df	1b+	2b+	S+
			
	n/46	p-value (%)	n/110	p-value (%)							n/39	p-value (%)	n/117	p-value (%)						
*Oesophagostomum* spp.	33	71.7	67	60.9	0.21	0.65	1	+		-	22	56.41	78	66.6	0.16	0.68	1		+	-
*Ancylostoma* spp.	2	4.3	15	13.6	1.6	0.21	1		+	-	7	17.94	10	8.5	1.31	0.25	1	+		-
*Trichostrongylus* spp.	4	8.6	24	21.8	2.05	0.15	1		+	-	9	23.07	19	16.2	0.31	0.6	1		+	-
*Strongyloides* spp.	0	0.0	7	6.3	16	0.20	1		+	-	7	17.94	0	0	15.1	0.0001	1	+		+
*Ascaris suum*	2	4.3	6	5.4	0	1	1		+	-	0	0	8	6.8	1.42	0.23	1		+	-
*Hyostrongylus* spp.	3	6.5	10	9.0	0.03	0.86	1		+	-	4	10.25	9	7.6	0.02	0.89	1	+		-
*Spirurida*	0	0.0	1	0.9	0	1	1		+	-	0	0	1	0.8	0	1	1		+	-
*Globocephalus* spp.	0	0.0	1	0.9	0	1	1		+	-	0	0	1	0.8	0	1	1		+	-
*Balantidium coli*	42	91.3	78	70.9	0.71	0.39	1	+		-	31	79.48	89	76	3^e-04^	0.98	1	+		-
*Isospora suis*	30	65.2	72	65.4	0	1	1		+	-	19	48.71	83	70.9	1.02	0.29	1		+	-

E+=Extensive having had the highest prevalence, SI+=Semi-intensive having had the highest prevalence, 1b+=1 per box having had the highest prevalence, 2b+=>2 per box having had the highest prevalence, S+=Indicating a statistically significant difference

Finally, for the physiological condition, our results revealed no significant difference between castrated and non-castrated individuals (Table-4). In addition, the comparison between pregnant and non-pregnant females revealed a significant difference only for *Hyostrongylus* spp. We noted that the prevalence of *Hyostrongylus* spp. was significantly higher in pregnant females (50%) than in non-pregnant (6.3%) (χ^2^=4.75, df=1, p=0.023) (Table-4).

**Table-4:** Prevalence of the types of parasites according to the physiological condition.

Parasites	Physiological condition																			

	Castrated		Non-castrated		Comparison						Non-pregnant		Pregnant			Comparison				
					
	n/17	p-value (%)	n/35	p-value (%)	χ^2^	p-value	df	C+	NC+	S+	n/47	p-value (%)	n/8	p-value (%)	χ^2^	p-value	df	NP+	P+	S+
*Oesophagostomum* spp.	14	82.3	19	54.2	0.46	0.5	1	+		-	18	38.2	7	87.5	1.25	0.26	1		+	-
*Ancylostoma* spp.	0	0	7	20	1.82	0.18	1		+	-	7	14.8	1	12.5	0	1	1	+		-
*Trichostrongylus* spp.	2	11.7	6.0	17.1	7^e-04^	0.98	1		+	-	5	10.3	3	60.0	1.21	0.27	1		+	-
*Strongyloides* spp.	0	0	3	8.5	0.30	0.58	1		+	-	2	4.2	0	0	0	1	1	+		-
*Ascaris suum*	2	11.7	2	5.7	0.025	0.87	1	+		-	3	6.3	0	0	0	1	1	+		-
*Hyostrongylus* spp.	1	5.8	2	5.7	0	1	1	+		-	3	6.3	4	50	4.75	0.023	1		+	+
*Globocephalus* spp.	0	0	0	0	-	-	-			-	1	2.1	0	0	0	1	1	+		-
*Balantidium coli*	12	70.5	29	82.8	0.016	0.89	1		+	-	37	78.7	7	87.5	0	1	1		+	-
*Isospora suis*	14	82.3	21	60.0	0.22	0.64	1	+		-	28	59.5	4	50	0	1	1	+		-

C+=Castrated having had the highest prevalence, NC+=Non-castrated having had the highest prevalence, NP+=Non-pregnant having had the highest prevalence, P+=Pregnant having had the highest prevalence, S+=Indicating a statistically significant difference

## Discussion

Several studies have reported a wide variety of gastrointestinal parasites in farmed pigs in Africa [8,16,17]. These studies on the interactions between parasites and their hosts and on the dynamics of transmission in different environments are indispensable as they serve as an indicator for the improvement of the health conditions of farmed animals. In this study, analyses were conducted on a set of 156 fecal samples taken from pigs from nine known farms in the Haut-Ogooué Province. All gastrointestinal parasites found in this study had all previously been identified in pigs.

Regarding the diversity of gastrointestinal parasites, ten types of parasites were identified, of which eight belong to the helminths and two to the protozoa. For each type of parasite, infestation rates were variable. However, for five of them, overall prevalence of infestation was higher than those of other parasites, namely, *Oesophagostomum* spp., *Ancylostoma* spp., *Trichostrongylus* spp., *B. coli*, and *I. suis*. Similar results have been found in numerous studies that have reported in pigs the identification of parasitic genera such as *Strongyloides*, *Ascaris*, *Oesophagostomum*, *Hyostrongylus*, *Ancylostoma*, *Balantidium*, and *Isospora* in India [18] and Nigeria [17]. These parasites are known to be common in farms and could be the cause of asymptomatic or subclinical infections [19], some of which could have deleterious effects on the health of animals or have a negative impact on the quality of the food product [11,20,21].

The overall prevalence of infestation (98.7%) observed in our study was higher than those found in previous studies that reported prevalence of 51.1% in India [22], 91% in Burkina Faso [8], 100% in Nigeria [23], and 79.2% in South Africa [24] in pigs. Our results, like those of the above-mentioned studies, show and suggest that pigs are infested with high levels by gastrointestinal parasites and this could be justified by the poor hygiene and rearing conditions observed in the most farms [25], the climatic conditions in the study area and the mode of transmission of these parasites. Indeed, for most gastrointestinal parasites, the transmission is direct or maybe through the ingestion of eggs by contaminated food consumption [26]. In addition, it has been shown that the conditions and environment in which animals are kept could influence their level of infestation [8,27]. In most of the farms studied, the animals’ boxes are not regularly cleaned, animal deworming is not regular or nonexistent, and the animals are very often underfed or poorly fed. In addition, this high prevalence could be associated with the climatic conditions encountered in the province during the study period; the majority of the samples have been taken during the high rainfall season characterized by high temperatures and high humidity. These are all conditions that are favorable to the development of free infestations [25].

For the analysis of the factors that can influence the level of parasite infestation in pigs, we have considered some factors such as sex, age, breed, breeding system, number of individuals per box, physiological condition, and the general body condition of animals. With respect to sex of animals, excepted one parasite (*B. coli*), no significant difference was observed between males and females. Moreover, infestation rates were higher in females for six of the ten identified parasites. High prevalence of infestation with *B. coli* and large diversity parasites observed in female pigs than males could be explained by alteration of some parameters such as physiological status or reproductive function (pregnancy, parturition, and lactation) which could weakening of organism or activities of immune system during these periods and predispose them to different parasitic infections like suggested in previous [24,28-30]. Furthermore, other justification could be the fact that animals are not well monitored or fed. However, our results corroborate those from a previous study reporting a higher level of infestation in female pigs than in males in Burkina Faso [8]. Indeed, many studies noticed this observation [8,24,31]. Moreover, it was recognized that females are generally more prone to helminth infestation than males during late pregnancy and lactation. We think that this observation could be attributed to hormonal changes at this time that lower their resistance to nematodes resulting in the establishment of higher worm burdens than in males [31,32]. Based on this result, sex could play an important role in the level of infestation in pigs.

Age could also influence parasite infestations. Indeed, our results show that six of the ten identified parasites were more prevalent in young pigs than in adults. This observation is supported by previous studies that have reported that age can influence the level of infestation of pigs [5,10,24,33]. Furthermore, the fact that adults have low rates of infestation compared to young could be explained by the establishment of immunity after the first infestation [8]. High infestation rates observed in young could be explained by the fact that their immune systems are not as effective as those of an adult who has acquired a more effective immune memory over time [34]. However, the higher level of *Hyostrongylus* infestation found in adults may be a consequence of the low immunogenicity of this parasite among adults’ pigs and/or overexposure of adults to these parasites [34,35].

The infestation level was higher in semi-intensive breeding. Indeed, in this type of breeding, the prevalence was higher for eight parasites, while in the extensive system, only two kinds of parasites had a higher prevalence. This result is consistent with literature that suggests that the range and intensity of infestation of nematode species depend on the type of system used [8,27]. In addition, infestation levels appear to be higher in imported breeds than in local but did not show a significant difference for any of the parasites identified in this study. Our results do not agree with those of Aiyedun and Oludairo [36] in Nigeria, who reported a higher prevalence among local breeds than those imported. However, imported breeds are most often reared intensively or semi-intensively and these breeding systems have been shown to promote the spread of parasites [37]. Thus, the imported breeds would be more vulnerable to local parasites, hence the highlighting of the high prevalence of certain gastrointestinal parasites in the latter. In addition, the low levels of infestation in local races could be due to they have acquired strong immunity to parasitic infestations due to recurrent infestations, like described in small ruminants [38].

Finally, for other factors such as the physiological condition and the general body condition of the animal, no significant difference was observed in our study. For the general condition, the highest prevalence was observed in animals in overweight compared to the thinned pigs. These results corroborate those of Batiebo [29] in Burkina Faso who found that overweight pigs were more infested than thinned pigs. Thus, observation of the high prevalence of parasite in overweight animals could be due to contaminated food or sullied with eggs of parasites [39]. Indeed, if the administered diet is poorly preserved or contaminated with parasite eggs, it is possible that these animals have a higher infestation than those with undernutrition [39]. However, it should be noted that parasitism affects the general body condition by causing stunting in young and weight loss [9]. For the physiological condition, no significant difference was observed between castrated and non-castrated pigs. However, the comparison between pregnant and non-pregnant revealed a statistically significant difference for one parasite, *Hyostrongylus* spp., which was more prevalent in pregnant females. This parasite is known to manifest lower immunogenicity in breeding pigs [35], in addition, the pregnancy period could have a favorable impact in parasitism because it alters or weak immunity. Thus, these different situations could explain the high level of *Hyostrongylus* found in pregnant. This parasite is known to have deleterious effects on the health of pregnant sows by causing lean sow syndrome [40]. Our observations join those of Morales *et al*. [41] who showed that gestation and castration play a role in the level of infestation. However, the insignificant differences in our observations concerning the assessment of the factors that may influence the level of infestation of pigs by intestinal parasites could be explained by the size of the population, which is low and does not allow us to appreciate exactly the influence of its various factors. We believe that additional studies that take into account a larger population of pigs would be necessary to invalidate or confirm all these observations.

## Conclusion

The preliminary study on the diversity and prevalence of gastrointestinal parasites in pigs has determined that at least ten parasites (*Ancylostoma* spp., *Oesophagostomum* spp., *A*. *suum*, *Strongyloides* spp., *Trichostrongylus* spp., *Hyostrongylus* spp., *Globocephalus* spp., *Spirurida*, *B. coli*, and *I*. *suis*) are present in pigs in Southeastern Gabon. The overall parasitic infestation rate (98.7%) of pigs by gastrointestinal parasites is very high. Furthermore, it has been shown that certain factors such as sex, age, and physiological condition may influence the diversity and level of infestation of animals by gastrointestinal parasites. Thus, for better prevention of parasitism in these farms, it would be interesting to implement health monitoring and to ensure good hygiene. Finally, further studies would be important to better evaluate the distribution of these parasites in Gabon and the involvement of these animals in the transmission cycle of parasitic zoonoses.

## Authors’ Contributions

GDM and JA conceived and designed the research. LB and GDM conducted the sample collection. LBK, LB, ICMK, and OBG carried out the parasitological analysis. LBK, LB, JO, and HT carried out the data analysis. GDM, LBK, and LB wrote the manuscript. HT reviewed the manuscript. All authors read and approved the final manuscript.
